# Identification of β-hematin inhibitors in the MMV Malaria Box

**DOI:** 10.1016/j.ijpddr.2015.05.003

**Published:** 2015-06-06

**Authors:** Kim Y. Fong, Rebecca D. Sandlin, David W. Wright

**Affiliations:** Department of Chemistry, Vanderbilt University, Station B 351822, Nashville, TN 37235, USA

**Keywords:** Antimalarial, Hemozoin, β-hematin, Biomineralization, *Plasmodium falciparum*, MMV Malaria Box

## Abstract

The Malaria Box, assembled by the Medicines for Malaria Venture, is a set of 400 structurally diverse, commercially available compounds with demonstrated activity against blood-stage *Plasmodium falciparum*. The compounds are a representative subset of the 20,000 *in vitro* antimalarials identified from the high-throughput screening efforts of St. Jude Children's Research Hospital (TN, USA), Novartis and GlaxoSmithKline. In addition, a small set of active compounds from commercially available libraries was added to this group, but it has not previously been published. Elucidation of the biochemical pathways on which these compounds act is a major challenge; therefore, access to these compounds has been made available free of charge to the investigator community. Here, the Malaria Box compounds were tested for activity against the formation of β-hematin, a synthetic form of the heme detoxification biomineral, hemozoin. Further, the mechanism of action of these compounds within the malaria parasite was explored. Ten of the Malaria Box compounds demonstrated significant inhibition of β-hematin formation. In this assay, dose–response data revealed IC_50_ values ranging from 8.7 to 22.7 μM for these hits, each of which is more potent than chloroquine (a known inhibitor of hemozoin formation). The *in vitro* antimalarial activity of these ten hits was confirmed in cultures of the chloroquine sensitive D6 strain of the parasite resulting in IC_50_ values of 135–2165 nM, followed by testing in the multidrug resistant strain, C235. Cultures of *P. falciparum* (D6) were then examined for their heme distribution following treatment with nine of the commercially available confirmed compounds, seven of which disrupted the hemozoin pathway.

## Highlights

• Ten of 400 Malaria Box compounds were found to be potent β-hematin inhibitors.
• We confirmed similar *in vitro* antimalarial activity to results from previous screens.
• 7 of the 9 commercially available hits were validated hemozoin inhibitors in culture.

## Graphical abstract

**Figure:**
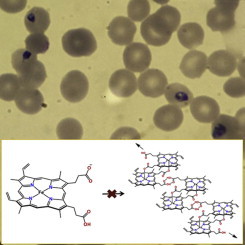

## 1 Introduction

It has been over fifty years since resistance to chloroquine (CQ) in *Plasmodium falciparum* was first reported, and since then the malaria parasite continues to rapidly develop resistance to current replacement therapeutics, including sulfadoxine-pyrimethamine and artemisinin combination therapies (Abdul-Ghani et al., 2013; Ashley et al., 2014; Wongsrichanalai et al., 2002). While pharmaceutical companies have lacked interest in developing new drugs for malaria, the advent of public-private partnerships (PPP's) has facilitated collaborative efforts between pharmaceutical companies with non-profit organizations and universities (Nwaka and Ridley, 2003). An exemplar PPP, Medicines for Malaria Venture (MMV), was established in 1999 to enable the discovery of new, effective and affordable antimalarial drugs. Notably, MMV supported the high-throughput screening (HTS) efforts of St. Jude Children's Research Hospital, Novartis and GlaxoSmithKline (GSK) to screen over 4 million compounds for *in vitro* antimalarial activity (Guiguemde et al., 2010; Plouffe et al., 2008; Gamo et al., 2010). Of these, over 20,000 compounds have been identified with potent *in vitro* antimalarial activity. Perhaps the most impressive aspect of this collaborative discovery effort is that the structures of these chemical starting points have been deposited in the ChEMBL neglected tropical diseases archive, an Open Access screening repository that allows researchers from around the world to access this data free of charge (https://www.ebi.ac.uk/chemblntd). To encourage the broader investigation of these compounds, MMV announced free access to the compounds of the Malaria Box – a set of 400 compounds selected from the 20,000 hits that are representative of the breadth of chemical diversity and predicted to be pharmacologically valid. While these compounds are potent *in vitro* antimalarials, all possible drug targets have not been explored. In this report, the Malaria Box compounds have been tested for inhibitory activity against the formation of β-hematin, the synthetic form of the heme-detoxification biomineral, hemozoin, followed by target validation in a parasite culture.

During the intraerythrocytic stage of the life cycle, the malaria parasite catabolizes host hemoglobin as its primary source of nutrition. This process occurs within the parasite's digestive food vacuole, an acidic organelle (pH ∼4.8) (Hayward et al., 2006). During the process of hemoglobin degradation, toxic free heme is released. Lacking the enzyme heme oxygenase used for heme-detoxification by most organisms, the parasite coverts the free heme into a non-toxic, insoluble crystal called hemozoin. Since the parasite catabolizes up to 80% of the erythrocyte's hemoglobin content, local concentrations of free heme could potentially reach 200–500 mM if hemozoin crystallization did not occur (Scholar and Pratt, 2000).

Hemozoin formation is mediated by neutral lipid bodies concentrated within the digestive food vacuole that serve as a reservoir for free heme (Hoang et al., 2010b; Pisciotta et al., 2007). These lipids were extracted from the parasite and shown to consist of a specific blend of mono- and di-acylglycerols (Pisciotta et al., 2007). Synthetic neutral lipid droplets (SNLDs) composed of the biologically relevant blend of neutral lipids were shown to be a kinetically competent site for the *in vitro* formation of β-hematin, with a crystallization half-life of <5 min (Hoang et al., 2010a, 2010b). In addition to accumulation of free heme within the SNLDs, molecular dynamic simulations have demonstrated that the lipophilic environment of the lipid body would serve to stabilize the hemozoin precursor dimer and that formation of hemozoin would be favored at the lipid/aqueous interface (de Villiers et al., 2007). These observations would suggest that inhibitors of hemozoin formation would interact with free heme either at the lipid/aqueous interface or within the neutral lipid body.

One of the most successful antimalarial ever developed, CQ, acts by inhibiting the formation of hemozoin (Egan et al., 1994; Sullivan et al., 1996). However, the parasite has developed resistance to certain quinoline antimalarials as a result of mutations in *Pf* CQ resistant transporter (*Pf*CRT), a membrane protein localized to the digestive food vacuole (Fidock et al., 2000). These mutations reduce the concentrations of CQ within the digestive food vacuole, acting as an efflux pump, thereby lowering the vacuolar CQ concentration and preventing the interactions with its target, heme/hemozoin. However, since resistance is not the result of changes in the pathway of hemozoin formation itself, this biomineralization process remains a valid drug target.

Recently, an HTS assay for β-hematin formation was developed (Carter et al., 2010) and utilized in a screen of 144,330 compounds (Sandlin et al., 2014). This assay utilized a low-cost, lipophilic micelle-forming detergent (Nonidet P-40) to mimic the neutral lipid bodies present within the digestive food vacuole. Within this screen there were 530 hits identified, and remarkably, 32% of the hits were also active in *in vitro* cultures of *P. falciparum*, inhibiting ≥90% parasitemia. Wright and coworkers argued that this retention of biological activity is due to the use of a biologically relevant mediator of crystallization, thus the *in vitro* β-hematin inhibition assay more closely recapitulates the chemistry of the digestive food vacuole. In these studies, the Malaria Box compounds were tested in the β-hematin formation assay to detect inhibitors of this crystallization process. Of the 400 tested, ten compounds were identified to have dose–response β-hematin inhibitory activity more potent than that of CQ.

While these compounds were shown to possess antimalarial and *in vitro* β-hematin inhibition activity, this drug target pathway must be validated within a parasite culture through an assay developed by Egan and coworkers (Combrinck et al., 2013). Within *P. falciparum* iron (III) protoporphyrin IX can be observed in three forms: hemoglobin, free heme, and hemozoin. Egan and coworkers showed that a dose response treatment of CQ, a known hemozoin inhibitor, resulted in a rise in free heme and a decline in hemozoin levels. In contrast, pyrimethamine, an antimalarial known to target folate biosynthesis without affecting hemozoin formation, resulted in a steady baseline level of free heme, while still causing parasite death. Quantification of these three heme species following a dose response treatment can be used to confirm the biological pathway targeted in culture. The nine commercially available β-hematin inhibitors found were subsequently examined for their levels of free heme and -seven were confirmed to act against this particular pathway in culture.

## 2 Materials and methods

### 2.1 Malaria Box compounds

The Malaria Box library of 400 compounds was provided by the MMV at a concentration of 10 mM in dimethyl sulfoxide (DMSO) in 384-well microtiter plates. Due to the small quantities of compound delivered in the Malaria Box, the additional testing was conducted using compounds purchased from ChemBridge and ChemDiv. While MMV666689 was identified as a potent β-hematin inhibitor in the screen, it did not undergo the additional testing due to lack of commercial availability.

### 2.2 β-Hematin formation assay

As previously described by Sandlin et al., 20 μL of water was added to clear 384-well flat bottom microtiter plates containing the Malaria Box compounds. Five microliters of 348 μM Nonidet P-40 detergent (NP-40, Shell Chemical Co. originated from Pierce Biotechnology, Rockford, IL) was added to each well to mediate the formation of β-hematin, followed by the addition of 7 μL of acetone to prevent heme precipitation. A 25 mM stock was prepared by dissolving hemin chloride in DMSO followed by one minute of sonication and filtration through a 0.22 μm PVDF membrane filter unit. A 228 μM hematin suspension was then prepared from the hemin stock in 2 M acetate buffer (pH 4.8). Twenty-five microliters of this hematin suspension was added to the plate before incubating for six hours in a shaking water bath at 37 °C (Sandlin et al., 2014). The assay was analyzed using the pyridine-ferrochrome method (Ncokazi and Egan, 2005). A solution of 50% pyridine, 20% acetone, water, and 200 mM HEPES buffer (pH 7.4) was added to each well (the final concentration of pyridine was 5% v/v). The plate was then put on a shaker for ten minutes. The absorbance of the resulting complex was measured at 405 nm on a BioTek Synergy H4 plate reader.

### 2.3 Identification of β-hematin inhibitors

A 125 nL volume of each Malaria Box compound (10 mM) was acoustically delivered to a 384-well plate using a Labcyte Echo 550 liquid handler. Duplicate plates were prepared and the final concentration of compound was ∼22 μM, near the IC_50_ of amodiaquine (AQ) (a known antimalarial β-hematin inhibitor) in this assay. Potentiator (IC_100_ of AQ) and vehicle (DMSO only, 0.22%) controls were added in an alternating checkerboard pattern to the first two and last two columns of each plate. The reagents described above for the β-hematin formation assay were added to each plate and incubated. Following addition of the pyridine solution, the percentage of free heme in each test well was determined relative to the positive and negative control wells. Any Malaria Box compound that inhibited >50% β-hematin formation was considered a hit.

### 2.4 Concentration response curves

Concentration response curves were determined for each hit using the β-hematin formation assay. A concentration range of 0–110 μM was tested. Sigmoidal dose–response curves were generated using GraphPad Prism v5.0 (March 7, 2007).

### 2.5 Malaria SYBR green 1-based fluorescence (MSF) assay

The *P. falciparum* strain D6 (Walter Reed Army Institute of Research [WRAIR]/Sierra Leone) was cultured using an adapted method by Trager and Jensen (Trager and Jensen, 1976). Inhibitors of β-hematin formation were tested in this CQ-sensitive strain of *P. falciparum* using a previously published method (Johnson et al., 2007) with modifications (Sandlin et al., 2014). Initial dose–response curves were established by testing compound concentrations from 0.03 to 23 μM with a final DMSO concentration of 0.25%. To ensure that DMSO did not interfere with parasite growth, a control plate was used containing wells with 0.25% DMSO and wells containing no DMSO. Concentration response curves were generated using GraphPad Prism v5.0. Subsequent testing with the hit compounds from this initial screen was done on both D6 and a multidrug-resistant strain of *P. falciparum* (C235, WRAIR). Dose response curves were conducted using the commercially purchased compounds with duplicate measurements.

### 2.6 Heme speciation assay

The heme speciation assay was conducted using a method previously described (Combrinck et al., 2013). A *P. falciparum* (D6) culture was sorbitol synchronized at the early ring stage before being evenly divided among 25 cm^2^ culture flasks and treated with the test compound at 0, 0.5, 1, and 2 times the IC_50_ value previously determined in the MSF assay. The cultures were then incubated at 37 °C and 5% O_2_, 5% CO_2_, 90% N_2_ until the late trophozoite stage was reached (∼32 h). At this time, saponin (0.05% final concentration) was used to selectively lyse the erythrocytes, leaving the trophozoites intact. The parasites were then lysed following a freeze–thaw cycle. The hemoglobin fraction present in the parasite was collected as the supernatant following the addition of 0.02M HEPES buffer (pH 7.4), 4% sodium dodecyl sulfate, and centrifugation. Pyridine was added to the resulting pellet to solubilize the free heme. The remaining substance consisted of hemozoin, which was solubilized by the addition of 0.1 M sodium hydroxide. The absorbance peak maximum at 405 nm was collected to quantify the percentages of each heme species (*Pf* hemoglobin, intercellular free heme, and hemozoin) present in each trophozoite culture. Parasite morphology was observed through microscopy analysis and percent survival was determined by SYBR Green I fluorescence.

### 2.7 Vacuolar accumulation ratio (VAR) and lipid accumulation ratio (LAR)

Vacuolar Accumulation Ratios (VARs) were calculated using the method of Krogstad and Lipid Accumulation Ratio (LAR) values were calculated using the model of Warhurst (Krogstad and Schlesinger, 1986; Warhurst et al., 2007). Here, a cytosol pH of 7.4 and vacuolar pH of 4.8 were used with the pK_a_ values generated by Marvin ChemAxon software to calculate VAR values. For LAR, logD values at a pH of 7.4 were also calculated with this software.

## 3 Results

**Fig. 1:**
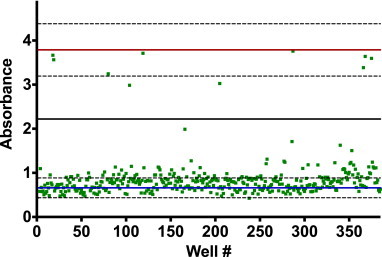
Identification of Malaria Box β-hematin inhibitors in the screen. The β-hematin formation assay identified ten potent inhibitors from the 400 Malaria Box compounds screened. The absorbance of test compounds is represented by green squares. The solid lines at 0.6 (blue) and 3.8 (red) represent the negative and positive controls, respectively, while the dashed lines represent three standard deviations of the respective control. The solid black line at 2.2 is the 50% inhibitory cutoff. Compounds above the 50% inhibitory threshold were considered hits. (For interpretation of the references to color in this figure legend, the reader is referred to the web version of this article.)

The Malaria Box compounds were screened for inhibitory activity with the β-hematin formation assay (Fig. 1). Ten hits were identified that inhibited >50% of crystallization relative to the IC_100_ of AQ and DMSO vehicle controls. Subsequent dose–response data indicated that the hits were very potent, with activities from 8.7 to 22.7 μM (the IC_50_ concentrations of AQ and CQ in this assay are 21.0 μM and 48.7 μM, respectively). In order to confirm that the integrity of the Malaria Box compounds received had not been compromised, the hits were tested in *in vitro* cultures of the CQ-sensitive D6 strain and multidrug-resistant C235 strain of *P. falciparum* (Table 1). The values obtained ranged from 135 to 2165 nM and 156–3469 nM, respectively, which are comparable to values reported by ChEMBL against the CQ-sensitive 3D7 strain.

The vacuolar accumulation ratio (VAR) is measured based on the pH difference between the cytosol (7.4) and the digestive food vacuole (4.8) in the parasite. The lipid accumulation ratio (LAR) is the ratio of drug found within the neutral lipid bodies of the digestive vacuole, the location of hemozoin formation. Calculated VARs and LARs for CQ (Table 2) are consistent with previously reported values and correlate with their β-hematin inhibitory activities (Warhurst et al., 2007). While the Malaria Box hit compounds do not possess similarly high VAR values, their LAR values indicate accumulation in lipophilic environments, explaining their high activity against this particular pathway. It is suggested that possessing high LAR values is especially important for activity against resistant parasite strains due to the lipophilic character of the drugs interacting with the hydrophobic channel of *Pf*CRT (Warhurst et al., 2007). This is evident with the resistance index values calculated (RI = IC_50_ of resistant strain (C235)/IC_50_ of sensitive strain (D6)). The relatively low RI values indicate these compounds are fairly potent against resistant strains, a vital component to novel antimalarials.

**Fig. 2:**
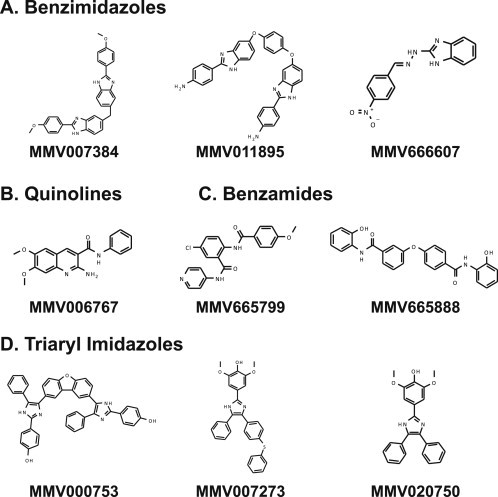
Main scaffolds identified as β-hematin inhibitors. Four scaffolds identified in the Malaria Box collection and their representative β-hematin inhibitors.

The Malaria Box compounds identified in the HTS assay are reflective of previously reported β-hematin scaffolds described in the literature (Fig. 2). Benzimidazoles were recognized as a potent inhibitor of β-hematin formation by Wright and coworkers (Sandlin et al., 2014) and likewise, three hits from the current study (MMV007384, MMV011895, and MMV666607) fall into this chemical scaffold. In another screen for β-hematin inhibition, Camacho et al. incorporated a nitrofuran into several benzimidazole-based structures and found efficacy on par with that of CQ (Camacho et al., 2011). In addition to this scaffold targeting the hemozoin pathway, these compounds have also been reported as potent antimalarials in both CQ-sensitive and CQ-resistant parasite strains. This is ideal in a novel antimalarial as *P. falciparum* drug resistance has quickly become widespread, causing major public health concerns. Therefore, if a compound is found active against resistant strains, it is more likely to move forward into the next round of testing.

The quinoline scaffold (MMV006767) was also identified in our screen of the Malaria Box compounds. Quinolines are perhaps the most well known and investigated β-hematin inhibiting scaffolds due to the potent activity of the quinoline derivative, CQ, against sensitive strains of *P. falciparum* (Ncokazi and Egan, 2005; Rush et al., 2009). Even with the widespread drug resistance against CQ, quinolines remain an effective scaffold since the mechanism of resistance is due to a gene mutation in *Pf*CRT and is unrelated to the mechanism of action (Sinha et al., 2014). Furthermore, it was found that drug resistance is compound specific, allowing quinoline derivatives to still be potential drug candidates (Ridley et al., 1996).

Two benzamide analogues (MMV665799 and MMV665888) were also identified as β-hematin inhibitors in the Malaria Box. In previous screens, similar benzamide compounds have been reported as inhibitors of β-hematin formation (Guiguemde et al., 2010; Sandlin et al., 2014). Furthermore, treatment with select benzamides have been shown to exhibit large intracellular free heme levels compared to other scaffolds, which can begin to provide insight into their exact mechanism of inhibiting hemozoin formation (Sandlin et al., 2014).

The triarylimidazole scaffold (MMV000753, MMV007273, and MMV020750) has been previously identified in the results of only one other β-hematin activity high-throughput screen (Sandlin et al., 2014). The remaining hit compound from this screen, MMV666689, is quite similar to the triarylimidazole scaffold with some activity against late-stage gametocytes (Sun et al., 2014).

**Fig. 3:**
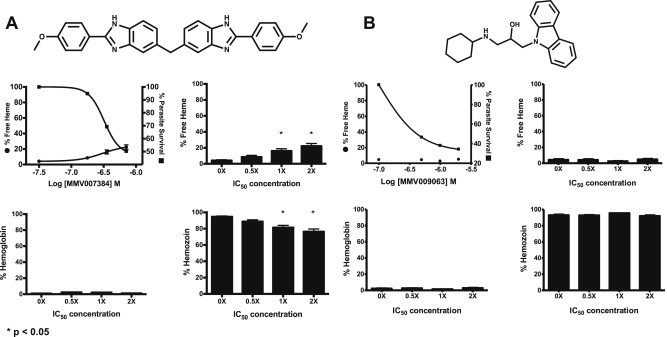
Target validation of hit compounds by comparing heme distribution following treatment. Distribution of heme species following treatment with a Malaria Box β-hematin inhibitor, MMV007384 (A) and a non-inhibitor, MMV009063 (B). Both compounds were found to have activity against D6 *P. falciparum* in the MSF SYBR Green I Fluorescence based assay and their dose response curves are shown. Statistical significance was calculated using a two-tailed unpaired t-test with p < 0.05.

The nine commercially available compounds underwent target validation within a culture of *P. falciparum* (D6) (Table 1). Following a dose response treatment of the β-hematin inhibitors, the changes in the distribution of iron (III) protoporphyrin IX was observed within *Pf* hemoglobin, free heme, and hemozoin. Seven of the nine antimalarial β-hematin inhibitors tested were found to perturb the hemozoin formation pathway in a parasite culture. This was shown through a significant (p < 0.05) increase in free heme levels from baseline, corresponding to decreases in hemozoin and parasite survival (Fig. 3). Parasite morphology by microscopy analysis confirmed that the control culture was indeed collected as late trophozoites. Increasing concentrations of drug treatment resulted in altered parasite morphology, demonstrating visually that they were no longer viable (Supplementary Figure 1). MMV009063 was shown to have *in vitro* antimalarial activity (IC_50_ = 610 ± 81 nM), but did not inhibit β-hematin formation. Therefore in addition to pyrimethamine (a non-hemozoin inhibitor) (Supplementary Figure 2), this compound was used as a negative control for target validation. A culture treated in a dose response manner with MMV009063 resulted in decreased parasite viability, while the free heme levels remained unchanged from basal levels, indicating it is acting upon a biological pathway other than hemozoin formation. By observing the distribution of iron (III) protoporphyrin IX following drug treatment, we can now focus on these hits that have been validated as hemozoin inhibitors in culture.

## 4 Discussion

Currently, most antimalarials only treat the blood stage of the parasite life cycle, as this is where it is most pathogenic resulting in febrile symptoms. However, if this disease is going to be eradicated, then treatment must also target the asymptomatic stages, including the liver and sexual stages in both drug sensitive and resistant strains (Delves et al., 2012). Recently, combination therapies have been used to combat challenges with drug resistance. This strategy could also allow for drugs to target separate pathways, which will increase its efficacy and help prevent recrudescence (Vathsala et al., 2012). Thus, both target based and phenotypic screening can narrow the focus towards wholly efficacious lead compounds.

The Malaria Box is the first open access library of compounds offered free of charge to the malaria research community. There are several advantages to the development of this library. Most importantly, open access to the Malaria Box increases the availability of these compounds to the malaria research community, which will facilitate target-elucidation. The process of target-elucidation is expensive and time-consuming, but is necessary in order to synthetically increase the affinity of the lead compound for its target. Since the Malaria Box is a reasonably manageable sampling of high-priority compounds, laboratories with expertise of specific biochemical pathways can quickly identify compounds that act on that target. In our screen for β-hematin inhibitors, we observed high potency within four scaffolds, consisting of ten hits.

### 4.1 Benzimidazoles

The most promising compound found in our screen of the Malaria Box set of compounds was MMV007384 as it was validated to be a β-hematin inhibitor and additionally reported active in both the asexual and sexual stages of the intraerythrocytic life cycle. This benzimidazole was reported to be a potent inhibitor of both early- and late-stage gametocytes, making it a more ideal malaria drug candidate (Duffy and Avery, 2013; Lucantoni et al., 2013; Bowman et al., 2014; Sun et al., 2014).

Another compound within this scaffold (MMV666607) is structurally similar to five of the MMV benzimidazoles in the study by Bowman et al. (MMV000248, MMV000444, MMV000445, MMV007384, MMV396723, MMV665902) as they each contain a guanidine moiety. However, they found that the structural requirements for gametocyticidal activity are rather strict, in that four of the five mentioned benzimidazole compounds didn't possess gametocytocidal activity despite some containing a secondary alcohol on the linker or similar tertiary amine moieties. MMV000248, MMV011895, and MMV666607 each contain activity against early- and late-stage gametocytes; (Lucantoni et al., 2013; Sun et al., 2014; Duffy and Avery, 2013) therefore, this scaffold space should still be explored further for its potential to target multiple stages of the parasite life cycle.

Benzimidazoles have also been screened against the pyrimidine biosynthetic enzyme dihydroorotate dehydrogenase (DHODH), but were found to have low potency (IC_50_ > 27 μM) and low selectivity between the parasite and human enzyme (Heikkila et al., 2007). The difference in activity levels between the two targets indicates that this class of compounds is more likely to affect a single asexual biochemical pathway *in vivo*. However, the concept of one drug targeting two pathways is of interest in the development of combination therapies.

### 4.2 Quinolines

In a recent screen with the Malaria Box collection for activity against gametocytes, quinoline compounds were found to be more potent against early-stage gametocytes compared to the asexual stage (Lucantoni et al., 2013). MMV665782 and MMV020500 exhibited a 9- and 7-fold reduced activity against the asexual stage of the 3D7 strain compared to early-stage gametocytes of the NF54 strain. However, these two specific Malaria Box compounds showed less than 20% inhibition of β-hematin formation in the present screen. In the search for a novel antimalarial, the most promising candidates include those that target the parasite in both the sexual and asexual stages.

### 4.3 Benzamides

The benzamide scaffold has also been shown to have inhibitory activity against *P. falciparum* DHODH (Heikkila et al., 2007). A common theme amongst the most potent members within this class of compounds is the presence of a halogenated substituent in the para position of the aniline moiety. Such substitutions play a role in CQ, as the chlorine, when placed in the 7 position, reportedly increases the binding affinity of the molecule to the hemozoin precursor (Vippagunta et al., 1999) as well as improve antiplasmodial activity overall (Lavrado et al., 2010).

### 4.4 Triaryl imidazoles

The MMV Malaria Box compounds were screened for early-stage gametocytocidal activity using a luciferase-based assay (Lucantoni et al., 2013). Two of the triaryl imidazole β-hematin inhibitors (MMV007273 and MMV000753) also contained activity against NF54 early-stage gametocytes with IC_50_ values of 1.361 and 0.627 μM, respectively. This scaffold should be pursued further as imidazoles have been found in many biologically active compounds including antimicrobial, antitumoral, and antiprotozoal (Rani et al., 2013).

### 4.5 Conclusions

In the search for a novel antimalarial, it is promising that these scaffolds contribute to multiple drug target pathways within *P. falciparum*. Due to the increasing drug resistance, a successful treatment will consist of a combination therapy, where each drug would target a distinct mechanism. This method of treatment is being used with artemisinin-based combination therapies, which are currently the most effective antimalarial medicine (WHO). Discovering a single compound that could simultaneously inhibit two biological mechanisms could decrease the cost and allow for a simpler treatment plan overall. Using combination therapies, such as a hemozoin inhibitor with an antifolate, will help prevent further development of resistance.

Reported here is the first published β-hematin inhibition specific data for the Malaria Box collection. Ten potent inhibitors of β-hematin formation have been identified from these compounds distributed by MMV, with seven of the nine commercially available having been validated for targeting the hemozoin formation pathway, resulting in an overall hit rate of 1.75%. The high validation rate indicates that the conditions of our *in vitro* β-hematin inhibition assay better represents the parasite environment of hemozoin formation as compared to other high-throughput β-hematin screens that only observed a hit rate of 0.1% (Rush et al., 2009). These seven inhibitors will undergo additional testing to determine pharmacokinetics of each compound and those with acceptable properties will continue on for testing of *in vivo* efficacy in the *Plasmodium berghei* mouse model.

## Conflicts of interest

The authors declare that they have no competing interests.

## Author's contributions

**Kim Y. Fong** performed experiments and was involved in the preparation of the manuscript. **Rebecca D. Sandlin** contributed to the conception of this work, performed experiments, and was involved in the preparation of the manuscript. **David W. Wright** contributed to the conception of this work, supervised experiments and was involved in the preparation of the manuscript.

## Figures and Tables

**Table 1:** Ten compounds from the Malaria Box identified to inhibit β-hematin formation and have antimalarial activity against *P. falciparum*. 50% inhibitory concentrations are listed against β-hematin inhibition, a CQ-sensitive strain (D6), and a multidrug-resistant strain (C235), along with target validation results with the heme speciation assay. CQ and pyrimethamine results are included as a positive and negative control, respectively, for known antimalarials.

Structure	MMV identifier	β-hematin IC_50_ (μM)	D6 IC_50_ MSF assay (nM)	C235 IC_50_ MSF assay (nM)	RI	Δ Free heme (%)
	MMV007384	10.6 ± 2.6	2165 ± 151^a^	3469 ± 24^a^	1.6	18
	MMV666607	22.7 ± 1.5	260 ± 23	458 ± 28	1.8	21
	MMV665799	16.0 ± 2.3	1639 ± 23	2765 ± 34	1.7	49
	MMV665888	13.1 ± 2.5	1410 ± 47	1305 ± 98	0.9	28
	MMV006767	14.8 ± 1.7	782 ± 35	1564 ± 77	2	35
	MMV007273	8.7 ± 2.1	262 ± 62	351 ± 2	1.3	21
	MMV000753	14.0 ± 4.9	1212 ± 4	1609 ± 77	1.3	21
	MMV020750	9.1 ± 2.1	366 ± 15	466 ± 22	1.3	0
	MMV011895	12.2 ± 2.0	135 ± 4	156 ± 6	1.2	0
	MMV666689	15.6 ± 2.5	465^b^	NT	NA	NT
	*MMV009063*	*Not Active*	*610 ± 81*	*856 ± 84*	*1.4*	*0*
	Chloroquine	48.7 ± 2.7	14 ± 1	48 ± 4	3.4	21
	*Pyrimethamine*	*Not Active*	*12 ± 2*	*NT*	*NA*	*0*

NA = Not applicable, NT = Not tested.

a Average mean and standard deviation of two replicates from commercially purchased compounds.

b Value from a single measurement, compound provided by MMV.

**Table 2:** Calculated vacuolar accumulation ratio (VAR) and lipid accumulation ratio (LAR) values of the ten identified hit compounds.

Structure	MMV identifier	Vacuolar Accumulation ratio	Lipid Accumulation ratio	Structure	MMV identifier	Vacuolar Accumulation ratio	Lipid Accumulation ratio
	MMV007384	25	1.6 × 10^6^		MMV007273	2	9.1 × 10^6^
	MMV666607	10	4.8 × 10^3^		MMV000753	6	2.5 × 10^9^
	MMV665799	7	2.4 × 10^3^		MMV020750	2	5.9 × 10^4^
	MMV665888	NA	1.0 × 10^5^		MMV011895	38	5.8 × 10^5^
	MMV006767	5	2.1 × 10^3^		MMV666689	1	1.1 × 10^6^

## Appendix A Supplementary data

Fig. S1 Morphological changes following drug treatment in the target validation assay Light microscopy images of the early ring stage where drug treatment occurs, followed by morphology of a mature trophozoite at the collection time. Morphology of cultures collected at 32 h following 2.5× drug treatment results in shrinkage, illustrating a non-viable parasite.

Fig. S2 Target validation of negative control known antimalarial, pyrimethamine Distribution of heme species followed by pyrimethamine, a known folate biosynthesis inhibitor. There is no significant (p < 0.05) difference between the 100 nM treated and control untreated culture.
