# Supplementary figures and images for: Identification of β-hematin inhibitors in the MMV Malaria Box
Source: Int J Parasitol Drugs Drug Resist. 2015 Jun 6;5(3):84–91. doi: 10.1016/j.ijpddr.2015.05.003 (PMC4486462; doi:10.1016/j.ijpddr.2015.05.003)

**Pre-treatment**

**32 hours post-treatment**

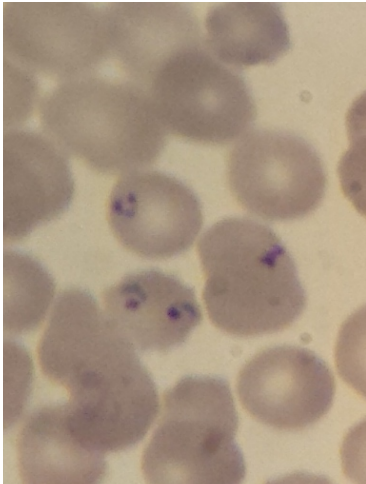

— **No treatment** —→

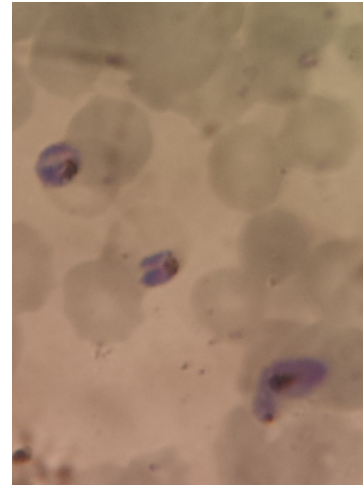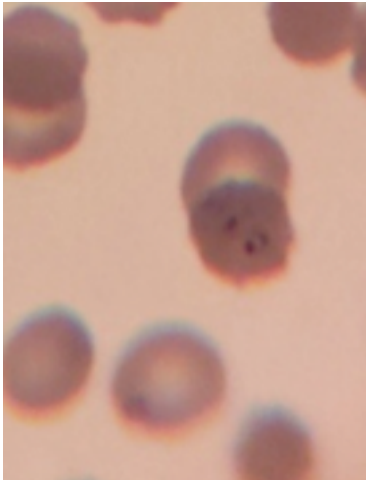

— **2x IC<sub>50</sub> treatment** —→

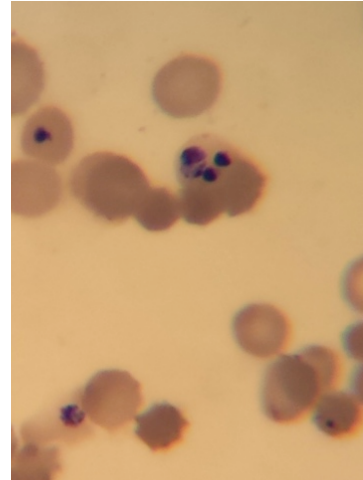

Supplement: Fig. S1 — Morphological changes following drug treatment in the target validation assay Light microscopy images of the early ring stage where drug treatment occurs, followed by morphology of a mature trophozoite at the collection time. Morphology of cultures collected at 32 h following 2.5× drug treatment results in shrinkage, illustrating a non-viable parasite. [file mmc1.pdf]

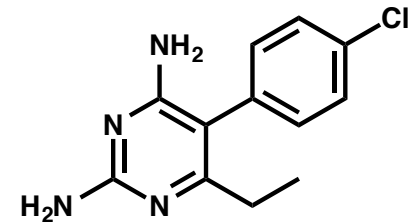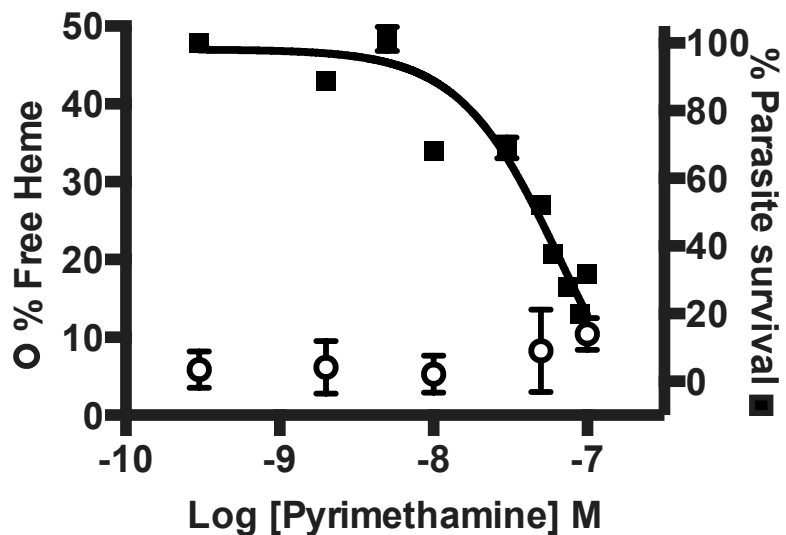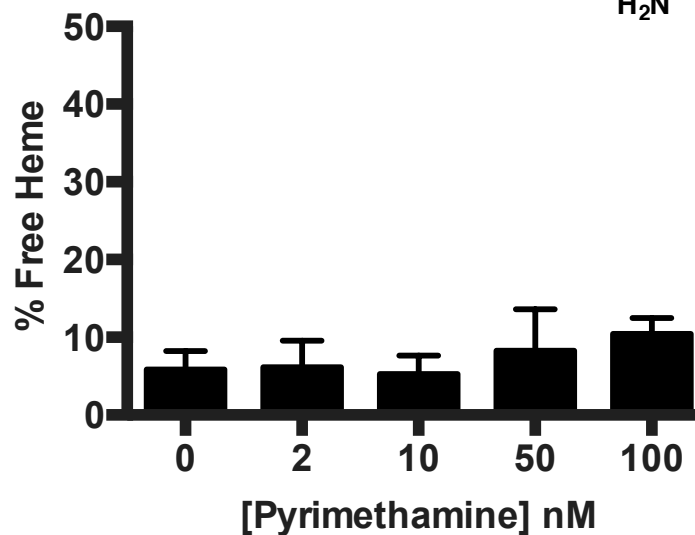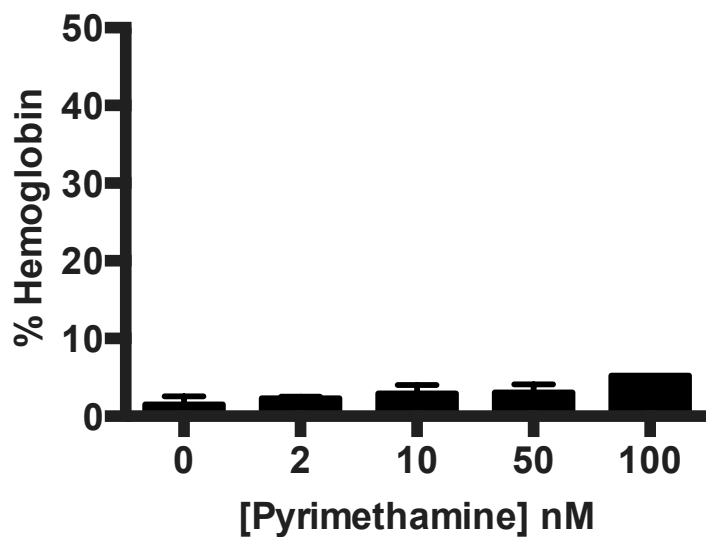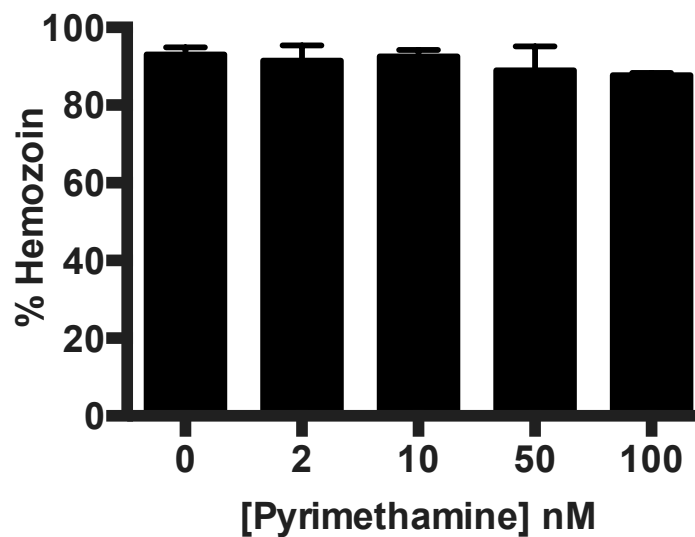

Supplement: Fig. S2 — Target validation of negative control known antimalarial, pyrimethamine Distribution of heme species followed by pyrimethamine, a known folate biosynthesis inhibitor. There is no significant (p < 0.05) difference between the 100 nM treated and control untreated culture. [file mmc2.pdf]
